# Littre’s Umbilical Hernia in a Child: A Case Report and Scoping Review

**DOI:** 10.7759/cureus.60510

**Published:** 2024-05-17

**Authors:** Florent T Zeng, Cheikh Seye, Papa A Mbaye, Ndèye A Ndoye, Doudou Gueye, Ibrahima B Wellé, Youssouph Diedhiou, Gabriel Ngom

**Affiliations:** 1 Pediatric Surgery, Albert Royer National Children’s Hospital Center, Université Cheikh Anta Diop, Dakar, SEN; 2 Pediatric Surgery, Université Alioune Diop, Diourbel, SEN; 3 Pediatric Surgery, Albert Royer National Children's Hospital Center, Université Cheikh Anta Diop, Dakar, SEN

**Keywords:** review, pancreatic ectopic tissue, meckel's diverticulum, umbilical hernia, littre's hernia

## Abstract

Littre’s umbilical hernia (UH) is a rare disease, the third most common Littre hernia. Most case reports interest adult patients. We reported the case of a four-year-old girl with anemia and symptomatic UH, with an incidentally diagnosed Meckel’s diverticulum (MD) containing pancreatic ectopic tissue. We reviewed case reports on Littre’s umbilical hernia without a date or language restriction. Including our patient, 21 cases were reviewed, of whom 15 (71.4%) were adults and 13 (61.9%) were males. Complicated umbilical hernia occurred in 13 patients (61.9%) and symptomatic MD in two children (9.5%). Investigations preoperatively diagnosed two patients (9.5%). Eighteen patients (85.7%) underwent open surgery, Meckel’s diverticulum removal was performed in 18 patients (85.7%), and primary umbilical hernia repair was performed in 16 (76.2%). Ectopic tissue was present in four patients (19.1%), and long-term outcomes were excellent in all patients.

## Introduction

Umbilical hernia (UH) is a common condition in pediatric surgical practice. Most pediatric UH is congenital, resulting from a faulty closure of the umbilical ring in the linea alba. In adults, UH is mainly acquired in populations with increased intra-abdominal pressure [1]. Congenital UH can close itself with the child aging. Therefore, for asymptomatic cases, expectative management is advised for four to five years [2]. However, symptomatic or complicated pediatric UH must undergo surgical repair [3].

The hernia sac may be empty during surgical repair or contain the greater omentum, small bowel, colon, or, surprisingly, Meckel’s diverticulum (MD), a remnant of the omphalomesenteric duct [3]. Meckel’s diverticulum has an incidence of 2% and is mostly asymptomatic. In 1700, Alexis de Littre reported an inguinal hernia containing an MD; then, a hernia sac containing an MD is referred to as a Littre hernia [4]. This is mainly encountered in femoral and inguinal hernias and rarely in umbilical, obturator, Spiegel, or incisional hernias [5,6].

Littre’s umbilical hernia (LUH), defined as the presence of an MD in a UH sac, is a rare condition; few publications exist on the subject. Preoperative diagnosis of LUH is exceptional; it is usually incidental during UH repair [6]. Its management depends on the management of the contained MD. Symptomatic MD (presenting with bleeding, inflammation, perforation, obstruction, or cancerization) should be ablated. However, controversies persist regarding the management of incidental MD, as some authors recommend no resection when others recommend it [7,8].

This study reports a LUH in an African girl. Additionally, to provide a comprehensive review of the circumstances of diagnosis, management, and outcomes of LUH, we conducted a scoping review of published case reports.

## Case presentation

A four-year-old girl consulted our hospital for umbilical swelling. She had no specific medical or surgical history. The umbilical swelling evolved since birth, with no tendency to spontaneous closure. Parents reported episodes of umbilical pain for months but no bloody or black stools. No pallor was noticed on examination, and vitals were within normal range. Examination showed an umbilical swelling, impulsive to cough, and reducible, with an umbilical defect estimated to be 3 cm. She was diagnosed with symptomatic UH (hernia with intermittent pain not linked to strangulation or incarceration), and the patient was planned for elective UH repair. Preoperative biology revealed microcytic hypochromic anemia (hemoglobin = 8 g/dL).

**Figure 1:**
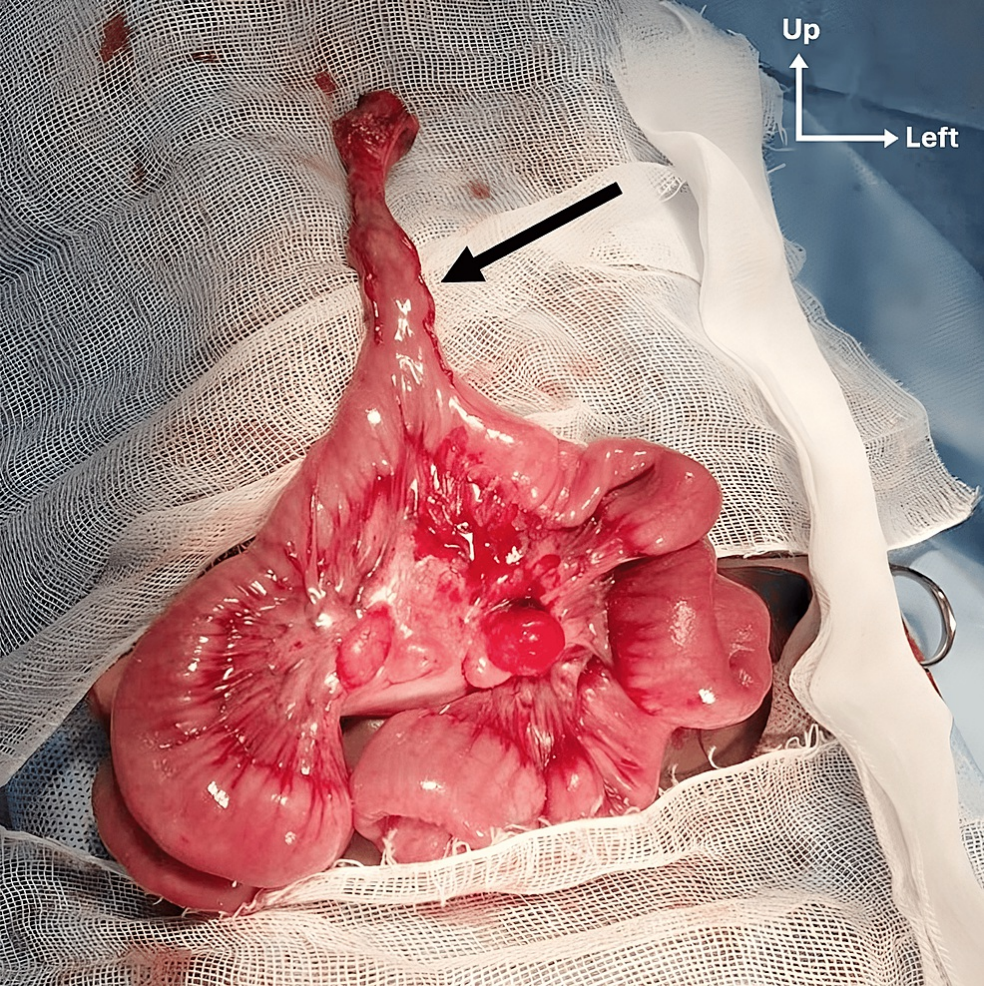
Intraoperative findings. Exposition of the MD (black arrow) with the bearing intestinal loop. MD: Meckel’s diverticulum.

The surgical intervention was carried out with a supra umbilical incision and dissection up to the UH sac. After its opening, an inflamed MD was found, with fibrous bands fixing it to the tip of the sac. The MD measured 1.5 cm in width at its basis and 4 cm in length (Figure 1). It was located 60 cm from the ileocecal junction (ICJ). In view of the patient’s age, MD length, and patient’s microcytic anemia, which made us suspect occult bleeding MD due to ectopic tissue (ET), a segmental resection of the ileal bearing loop was performed, 5 cm on both sides from the MD. End-to-end single-layer anastomosis was performed using 3/0 polyglactine continuous sutures. The umbilical defect was primarily repaired using 0 polyglactin continuous sutures and subcutaneous tissue approximated with 4/0 polyglactin interrupted sutures.

Postoperatively, the patient received intravenous analgesics and antibiotics (for three days). Oral feeding started on day two, after the resumption of bowel movements. The postoperative course was uneventful, and the patient was discharged on day five. Anatomopathological examination of the resected bowel confirmed a true diverticulum with the presence of ectopic pancreatic tissue at its basis, hemorrhagic changes and polynuclear infiltration. Ten months postoperatively, the patient has no complaints, and the physical examination is normal. The latest full blood count (FBC) did not depict any anomaly.

## Discussion

**Figure 2:**
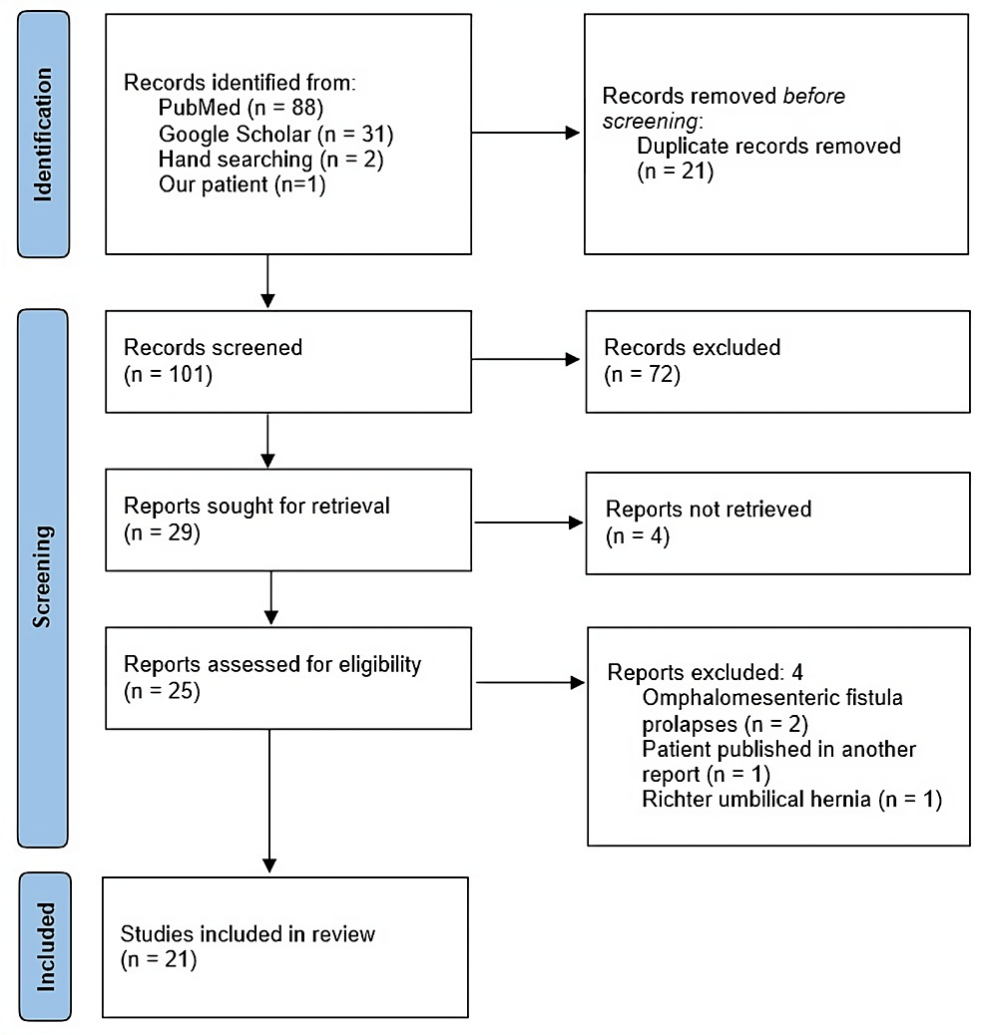
PRISMA flowchart. Depiction of the search strategy and study selection. PRISMA: Preferred Reporting Items for Systematic Reviews and Meta-Analyses.

Through MEDLINE/PubMed and Google Scholar, on 31 December 2023, a search was performed using the following search strategy: in PubMed: ((Meckel's diverticulum) AND (Umbilical hernia)) OR (Littre Umbilical hernia). In Google Scholar, two combined searches were used: ‘‘allintitle: Littre's Umbilical hernia’’ and ‘‘allintitle: Meckel's Diverticulum Umbilical hernia’’. Eligible studies reported LHU with no date, language, or age restriction. Study selection was performed using Rayyan® software (Rayyan Systems, Cambridge, MA, USA) following the Preferred Reporting Items for Systematic Reviews and Meta-Analyses (PRISMA) guidelines for scoping review [9]. The PRISMA flowchart is detailed in Figure 2.

Variables studied in the review were sociodemographic, diagnostic, therapeutic, anatomopathological, and outcomes. The analysis was carried out with IBM SPSS Statistics for Windows, Version 20 (Released 2011; IBM Corp., Armonk, New York, USA). For continuous variables, results will be presented as means or median according to the distribution of the variable, with normality being verified by the Kolmogorov-Smirnov test (p < 0.05). Categorical parameters will be presented as frequencies. Seeing the review nature of this study, ethical approval was waived by the institutional board.

After selection, 21 patients (including ours) were considered in the review [10-29]. There were 15 adults (71.4%) and six children (28.6%), with a mean age of 38.2 (±27) years, ranging from 24 weeks to 85 years. There were 13 males and eight females, with a sex ratio of 1.6:1. Table 1 details additional sociodemographic parameters of the included patients.

**Table 1:** Reports included in the review. UK: United Kingdom, USA: United States of America.

Authors	Country	Number of patients
Papadopoulos, 1915 [10]	UK	1
Castleden, 1970 [11]	Australia	1
Komlatsè et al., 2009 [12]	Togo	1
Sengul et al., 2010 [13]	Türkiye	1
Kurnicki et al., 2011 [14]	Poland	1
Augestad et al., 2012 [15]	Norway	1
Kibil et al., 2012 [16]	Poland	1
Naveed et al., 2012 [17]	USA	1
Noukpozounkou et al., 2018 [18]	Benin	1
Ariyoshi et al., 2020 [19]	Japan	1
Evola et al., 2021 [20]	Italy	1
Krishnaswamy et al., 2021 [21]	Australia	1
Matias et al., 2021 [22]	UK	1
Das et al., 2022 [23]	India	1
Nakamura, 2022 [24]	Japan	1
Ali et al., 2023 [25]	Somalia	1
Bishop et al., 2023 [26]	USA	1
Ghorishi et al., 2023 [27]	USA	1
Khalifa et al., 2023 [28]	Tunisia	1
Prakash et al., 2020 [29]	India	1
Our report, 2024	Senegal	1

Symptoms were present in 18 patients (85.7%), and abdominal or umbilical pain was reported in all symptomatic patients. Vomiting was found in eight of the 18 symptomatic patients (44.4%), constipation in three of them (16.7%), and nausea in two (11.1%). The following symptoms were reported once (5.9%): abdominal bloating, blood per rectum, dysuria, umbilical effusion, and redness. A recurrence of symptoms was reported in six patients (28.6%). The duration of symptoms was reported in 18 patients; the median was 2 (1.2-192) days, ranging from 0.5 to 730 days.

**Figure 3:**
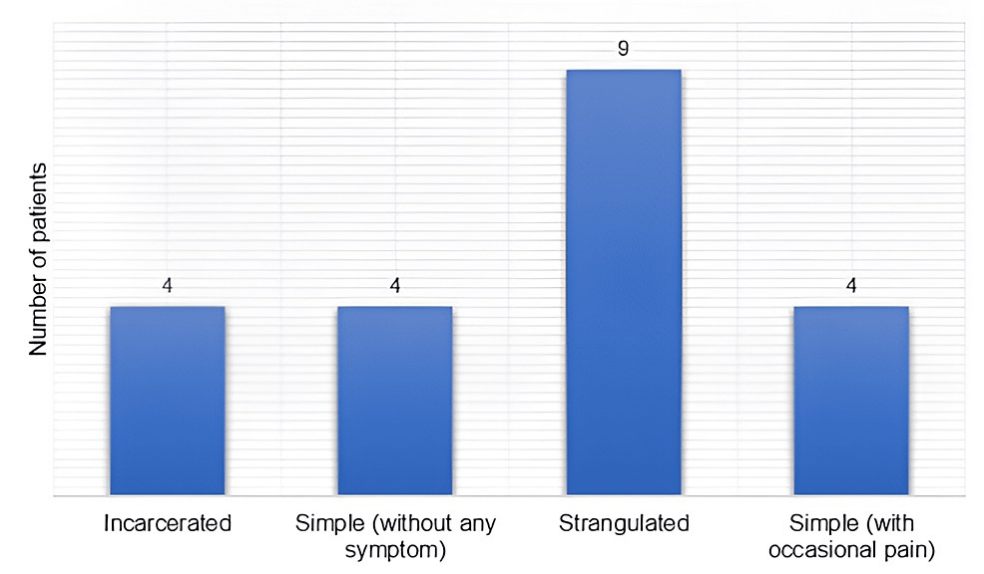
Diagnosis of umbilical hernia. Strangulation was in nine patients (43%), and other presentations were in four patients (19%) each.

On the physical examination, pallor was found in two patients (9.5%) and fever in four (19%). Abdominal distension was reported in two patients (9.5%) and umbilical swelling in 18 (85.7%). The size of the umbilical defect was reported in 13 patients, ranging from 1 to 7 cm, with a median of 3 (3-3.6) cm. Nine of the UH (43%) were strangulated; the diagnosis of UH is depicted in Figure 3. Two patients (10%), all children, had symptomatic MD with blood per rectum.

**Figure 4:**
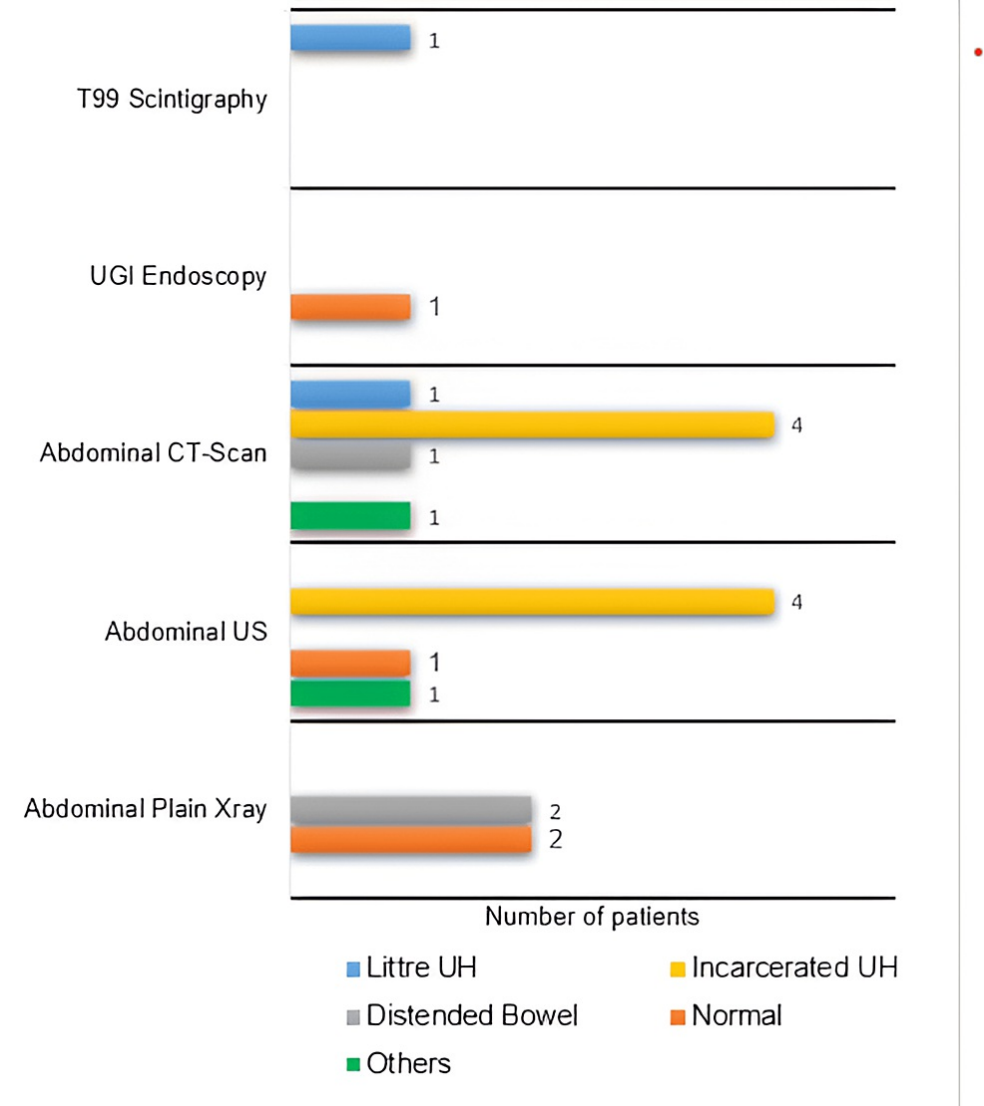
Findings of imaging investigation. Littre UH was preoperatively diagnosed in two patients by T99 scintigraphy and abdominal CT scan. Others: pneumoperitoneum (for CT patients) and inconclusive (for US patients). CT: computed tomography, T99: Technetium 99, UGI: upper gastrointestinal, UH: umbilical hernia, US: ultrasound.

In 13 patients (61.9%), 19 imaging exams were requested: plain abdominal X-ray in 4 (21%), abdominal ultrasound (US) in 6 (31.2%), abdominal computed tomography scan (CT scan) in 7 (31.2%), upper gastrointestinal tract endoscopy and Technetium 99 scintigraphy in 1 (5.3%) each. Details of their findings are depicted in Figure 4. The full blood count (FBC) was requested in 11 patients (52.4%), and three of them (27.3%) had anemia, while hyperleukocytosis was found in 4 (36.4%).

Concerning management, two patients (9.5%) with perforated MD required resuscitation before surgery, which was emergent in 14 patients (66.7%), semi-elective in 2 (9.5%), and elective in 5 (23.8%). Open surgery was performed in 18 patients (85.7%), and laparoscopy was performed in 3 (14.3%). The distance from the ICJ to the MD was reported in eight patients and ranged from 12 to 91 cm; its mean was 56 (±30.1) cm. The MD length was stated in 12 patients and ranged from 1.5 to 7 cm, with a mean of 4.7 (±1.8) cm. Details of operative findings are given in Table 2. The umbilical hernia was repaired primarily in 16 patients (76.2%), with synthetic material in 3 (14.3%) patients, and using the mixed method in 2 (9.5%). For primary repair and mixed method (18 patients), interrupted sutures were used in 4 (22.2%), continuous sutures in 2 (11.1%), and in 12 (66.7%) patients, the type of suture was not mentioned. Repair with synthetic material and mixed method (five patients), intraperitoneal mesh was used in three patients (60%), and meshrraphy in two patients (40%).

**Table 2:** Intraoperative findings and surgical indications for MD. ETEA: end-to-end anastomosis, MD: Meckel’s diverticulum, STSA: side-to-side anastomosis.

Parameters	Number	Percentage
Sac content		
MD alone	16	76.2
MD + great omentum	2	9.5
MD + small bowel	2	9.5
MD + small bowel + great omentum	1	4.8
MD gross aspect		
Normal	2	9.5
Abnormal	19	90.5
Inflammation	8	38
Distension	3	14.3
Necrosis	3	14.3
Inflammation + distension	2	9.5
Perforation	2	9.5
Ulceration	1	4.8
MD adherence to the sac		
Yes	9	42.9
No	12	57.1
MD iatrogenic injury		
Yes	2	9.5
No	19	90.5
MD surgical management		
Reintegrated	3	14.3
Segmental resection + ETEA	7	33.3
Segmental resection + STSA	1	4.8
Wedge resection + bowel suture	5	23.8
Diverticulectomy + bowel suture	5	23.8

**Figure 5:**
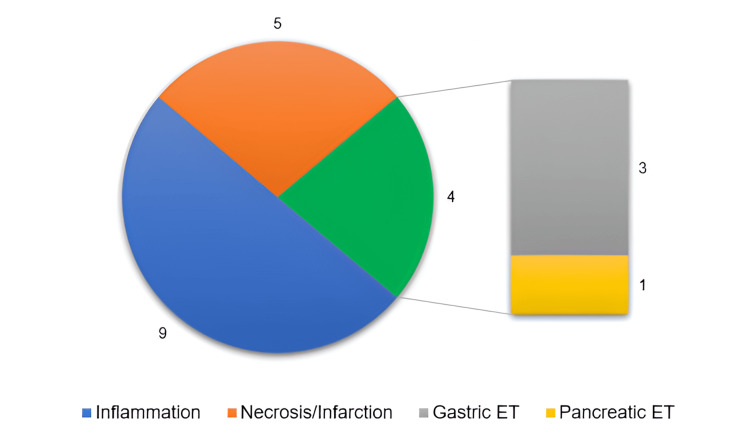
Anatomopathological findings. Ectopic tissue (green) was present four times, of which three were gastric (one diagnosed by T99 scintigraphy alone, with no anatomopathological report) and one pancreatic (our patient). ET: ectopic tissue.

In the early postoperative period, complications were reported in two patients (9.5%): superficial surgical site infection (SSI) in a patient whose UH was repaired with meshrraphy. The patient was treated without the need to remove sutures or the mesh. Another patient presented postoperative ileus, which regressed spontaneously. The time to discharge was reported in 16 patients and ranged from same day to 10 days, with a mean of 5.1 (±2.8) days. The anatomopathological results were reported in 17 patients, with 11 (64.7%) depicting microscopical anomalies, detailed in Figure 5.

An umbilical hernia from faulty closure of the umbilical ring (congenital UH) or increased intra-abdominal pressure (acquired UH). Congenital UH is frequent in African children, and acquired UH is primarily found in adults [1]. Depending on clinical presentation, UH may be simple (causing no symptoms apart from the usual bulging of a hernia), symptomatic (causing occasional umbilical pain, without any other symptoms), incarcerated, or strangulated (with pain, vomiting, constipation due to bowel entrapment within the hernial defect, with or without bowel ischemia) [3].

Due to umbilical proximity, the great omentum and the small intestine are more prompt to get through the umbilical defect. Exceptionally, 0.8% of UH may contain an MD [3], which is a remnant of the omphalomesenteric or vitello-intestinal duct [4]. In a normal situation, it regresses by the fifth week of gestation. When this fails, possible anomalies are omphalomesenteric sinus, omphalomesenteric cyst, omphalomesenteric fistula, MD, and Meckel band [4]. Meckel's diverticulum is the most frequent congenital anomaly of the GIT, reported up to 2.9% in the general population [30].

In 1700, the French surgeon Alexis de Littre reported a ‘‘new species’’ of hernia by incidentally finding an MD within an inguinal hernia [4]. Since then, the presence of MD in different hernias was reported: inguinal, femoral, umbilical, obturator, Spigelian, transthoracic, and incisional [5,6]. To our knowledge, LUH was first reported by Papadopoulos in 1914 [10]. It represents 11% of Littre's hernias [5,6]. In this review, 15% of patients had a flat umbilicus. Therefore, the clinician should always palpate the umbilicus to look for an umbilical defect in patients with abdominal complaints, especially in African children, where UH is common, and UH with flat umbilicus is not an exception [3]. In a third, UH was strangulated, with half occurring in children, all originating from Africa. Previously, authors reported frequent complications of pediatric UH in Africa compared to high-income countries (HICs) [31,32].

Meckel’s diverticulum can contain ectopic tissue (ET) [6]. In the included patients, all symptomatic MDs had ET. This is well described as ET is more common in symptomatic MD (41.5%) than in asymptomatic MD (11.4%). Gastric ET is the most frequent (44.3%), followed by pancreatic ET (8.6%) [30]. Exceptionally, carcinoid, duodenal, lipomatous, and mucocele ET were reported [33]. To our knowledge, we reported the first LUH with pancreatic ET.

A tenth of patients had symptomatic MD. In fact, only 4.2 to 6.3% of people with an MD will be symptomatic [8,30]. Symptoms are more common in males and younger patients (less than five to 10 years old) [6]. In this review, both symptomatic patients were less than five years old. The presentation includes bleeding, inflammation, perforation, obstruction, and cancerization. Bleeding occurs in the presence of gastric or pancreatic ET by ulceration of the normal adjacent intestinal mucosa due to highly acid or alkaline secretions of ET, respectively [34]. It manifests as painless blood per rectum, which may be massive or occult [35]. The two patients with bleeding MD both had an ET (gastric and pancreatic, respectively). Inflammation or Meckel’s diverticulitis results from the same mechanism as in acute appendicitis: an obstruction of its lumen [34]. The cause may be an enterolith, a foreign body, or rarely parasites. If not treated, inflammation will result in perforation and presents as acute peritonitis [34]. Obstruction secondary to MD can result from intussusception, with the MD acting as a lead-point, or from the segmental volvulus of the adjacent small bowel due to a fibrous band attaching the MD to the umbilicus [34]. In the mesenteric type of MD, obstruction can result from internal herniation [4]. Symptoms of MD are not specific. An inflamed MD can present as acute appendicitis, a reason why MD has been called the second appendix [34,36]. Consequently, most MDs are intraoperatively incidentally found during surgical exploration for acute abdomen [6].

Medical imaging allowed preoperative diagnosis in a tenth of patients. It is known that the abdominal US, plain X-ray, and CT scan rarely diagnose MD [30]. This must be fastidious for complicated UH due to viscera compression within the hernia sac [37]. Among the 17 patients with complicated UH who benefited from CT, only one was preoperatively diagnosed. Diagnosing a complicated hernia is clinical; the above imaging modalities are interesting in evaluating the degree of obstruction and bowel viability and may outline bowel perforation, thus giving the proper surgical indication [8]. However, when not available, they should not delay surgical exploration; bowel necrosis or perforation may meanwhile occur. Other explorations include angiography, which can display a bleeding MD, for which capsule endoscopy has shown promise. Technetium-99m pertechnetate scintigraphy, a proper diagnostic tool for ectopic gastric mucosa, will only diagnose MD with gastric ET [8]. This review allowed preoperative diagnosis in a patient with bleeding MD.

The surgical approach used in most of this review was open surgery. This may be linked to most patients presenting with complicated UH, a surgical emergency. In such a context, laparoscopy or a surgeon who can perform it is not always available, especially in resource-constrained settings [38]. During the sac opening, injury to the MD occurred in two patients. We emphasize the need for complicated UH surgery to be performed by or under close supervision of an experienced surgeon. The management of MD depends on whether it is symptomatic or not. Of the included patients, two were symptomatic and were resected. Among the 19 remaining patients, 16 were resected, and three were reintegrated. The authors agree that symptomatic MD should have been resected. However, the management of silent MD is highly controversial; some authors recommend systematic resection, while others contraindicate it [7,33,36]. The recommended attitude is to decide case by case, with resection performed in a single of the following situations: male patient, patient younger than 50 years, MD longer than 2 cm, and suspicion of ectopic tissue [33]. Considering this, only one of the three reintegrated MDs should not be resected, and one of the 19 resected MDs should be reintegrated. This depicts persisting variability in the management of incidental MD. In the complicated hernia context, it is natural that perforated and necrotic MD must be resected.

The resection modality of MD is variable. It encompasses simple diverticulectomy plus bowel suture, MD wedge resection plus bowel suture, segmental bowel resection of the bearing intestinal loop plus end-to-end anastomosis [33]. However, simple diverticulectomy may leave persistent ectopic tissue on the bearing segment [30]. Wedge resection may also leave ET, and its anastomosis can be a lead point for intestinal obstruction [4]. For the above reasons, many authors favor segmental resection, the most used technique in this review.

Pediatric UH is primarily treatable with sac resection and primary suture with purse string, continuous or interrupted suture with the same results [39]. All pediatric patients in this review benefited from primary repair. Primary repair is possible in adults, but some authors suggest prosthetic repair when the defect exceeds 3 cm [40]. However, in the context of emergency surgery, the mesh may not be available in resource-constrained settings, or surgeons may avoid prosthetic repair as it may be associated with SSI. A solution to the latter problem is to perform a two-step laparoscopic repair, with prosthetic repair on the second step [19].

Limitations

This review included 21 patients with LUH. However, some key elements were not mentioned in reports: size of the umbilical defect, distance from the MD to the ICJ, size of the MD, and type of sutures in UH primary repair. Including these parameters would be helpful to better analyze them.

## Conclusions

If unexplained microcytic anemia occurs in a child with umbilical hernia, occult blood per rectum should be investigated, and the possibility of an MD with ectopic tissue should be considered. In such cases, segmental bowel resection and end-to-end anastomosis provide excellent outcomes. Littre’s UH is a rare disease, and summarizing its presentation and management will help better understand it.
